# New Insights into Flavivirus Evolution, Taxonomy and Biogeographic History, Extended by Analysis of Canonical and Alternative Coding Sequences

**DOI:** 10.1371/journal.pone.0117849

**Published:** 2015-02-26

**Authors:** Gregory Moureau, Shelley Cook, Philippe Lemey, Antoine Nougairede, Naomi L. Forrester, Maxim Khasnatinov, Remi N. Charrel, Andrew E. Firth, Ernest A. Gould, Xavier de Lamballerie

**Affiliations:** 1 Aix Marseille Université, IRD French Institute of Research for Development, EHESP French School of Public Health, EPV UMR_D 190 Emergence des Pathologies Virales, Marseille, France; 2 Department of Life Sciences, Natural History Museum, Cromwell Road, London SW7 5BD, United Kingdom; 3 Department of Microbiology and Immunology, Rega Institute, KU Leuven, Minderbroedersstraat 10, 3000 Leuven, Belgium; 4 Institute for Human Infections and Immunity and Department of Pathology, University of Texas Medical Branch, Galveston, TX 77555, United States of America; 5 Centre for Ecology and Hydrology, Maclean Building, Benson Lane, Crowmarsh, Gifford, Wallingford, Oxfordshire, OX10, United Kingdom; 6 Division of Virology, Department of Pathology, University of Cambridge, Cambridge CB2 1QP, United Kingdom; Utah State University, UNITED STATES

## Abstract

To generate the most diverse phylogenetic dataset for the flaviviruses to date, we determined the genomic sequences and phylogenetic relationships of 14 flaviviruses, of which 10 are primarily associated with *Culex* spp. mosquitoes. We analyze these data, in conjunction with a comprehensive collection of flavivirus genomes, to characterize flavivirus evolutionary and biogeographic history in unprecedented detail and breadth. Based on the presumed introduction of yellow fever virus into the Americas via the transatlantic slave trade, we extrapolated a timescale for a relevant subset of flaviviruses whose evolutionary history, shows that different *Culex*-spp. associated flaviviruses have been introduced from the Old World to the New World on at least five separate occasions, with 2 different sets of factors likely to have contributed to the dispersal of the different viruses. We also discuss the significance of programmed ribosomal frameshifting in a central region of the polyprotein open reading frame in some mosquito-associated flaviviruses.

## INTRODUCTION

The flaviviruses constitute a fascinating group of diverse arboviruses that exhibit uniquely clear correlations between phylogenetic relationships and virus-vector-host interactions [1–4]. The genus *Flavivirus* includes an unusually large number of taxonomically recognised species (more than 50 at the present time, of which more than 40 are human pathogens) with a global distribution. The genus also includes a large and increasing number of unclassified or “tentative” species. Pathogenic mosquito- and/or tick-borne flaviviruses cause a variety of clinical diseases in a wide range of vertebrate species. These disease syndromes include mild/severe febrile illness, “flu-like” syndromes with a rash, or in other cases severe encephalitis or haemorrhagic disease. Dengue with / without warning signs and severe dengue, is the most devastating arboviral disease in tropical and, increasingly, sub-tropical areas of the world (300 to 400 million cases each year) [5]. Yellow fever virus and Japanese encephalitis virus also considerably contribute to the human flavivirus disease burden. Other flavivirus diseases, including West Nile encephalitis, Usutu encephalitis, Zika fever, Bagaza encephalitis and duck egg drop syndrome, are recognised as “emerging diseases”.

Flavivirus ecological networks are varied, complex, and poorly understood. Importantly, most of the natural pathogens are transmitted by arthropods (*i*.*e*. they are “arboviruses”). However, flaviviruses with no known vector (NKV), or that infect only insects (*i*.*e*., insect-specific flaviviruses—ISFVs), have also been identified. This remarkable diversity is associated with broad genetic variability, complex mechanisms of pathogenesis and intriguing virus/vector/host associations.

The taxonomy of the flaviviruses is constantly being updated to reflect newly-identified viruses and advances in analytical methods. Interestingly, the first mammalian viruses to be identified included 4 arboviruses, three of which were flaviviruses: louping ill virus, yellow fever virus and dengue virus [6]. This has had a deep and long-lasting influence on the development of virological research and more specifically, on taxonomy [7] and phylogeography.

With the publication of many new complete genomic flavivirus sequences [3,8] and the data generated in the current study, it is now timely and appropriate to re-examine the phylogenetic relationships in the context of flavivirus vector-host relationships, evolution and biogeographical characteristics.

The concept that the phylogenetic relationships of the tick-borne flaviviruses (TBFV) may correlate with their epidemiology, disease association and biogeography was first proposed in 1996 with the publication of the clinal evolution concept of the tick-borne encephalitic flaviviruses [9]. These relationships were then corroborated and extended by the inclusion of the mosquito-borne and non-vectored flaviviruses [1] but analyses were still based on a limited number of recognised flaviviruses and only partial gene sequence data (44 species based on the envelope gene). Subsequent analyses [3,8,10,11], using more extensive datasets improved our understanding of these virus-vector-host relationships in the context of their evolution and dispersal [2,12]. Additional flaviviruses including Lammi virus [13], N’goye virus [14], Alkhumra haemorrhagic fever virus [15], Usutu virus [16], New Mapoon virus [17], and Marisma mosquito virus [18] have subsequently been discovered. Together with the increasing numbers of documented species and strains of ISFV, that do not appear to be arboviruses [19–23], these discoveries have shed new light on our perception of the evolution and taxonomy of this complex genus.

Based on the flavivirus arthropod vectors and vertebrate hosts, current phylogenies recognise three major groups in addition to the ISFVs [1,3,8,10,13]: the tick-borne, mosquito-borne, and no known-vector flaviviruses (TBFV, MBFV and NKV respectively). The TBFV are sub-divided into pathogenic flaviviruses primarily associated with *Ixodes* spp., and apathogenic flaviviruses associated with *Ornithodorus* spp. ticks that primarily feed on or parasitize, seabirds. The mosquito-borne flaviviruses (MBFV) are sub-divided into those primarily associated with *Culex* spp. (ornithophilic) mosquitoes and those primarily associated with *Aedes* spp. (mammalophilic) mosquitoes. In contrast, the viruses with no known vectors (NKV) are divided into those primarily associated either with rodents or bats. These specific lineage NKVs are defined in the text as NKV^SL^. In addition, the three viruses (Sokoluk, Entebbe bat and Yokose virus) are exceptional in that they diverged with the MBFV but arthropod vectors have not been associated with these viruses. In other words, they diverged within the MBFV—*Aedes* spp.-associated clade but appear to have lost this mosquito association [10]. These NKV are defined in the text as NKV-like.

For further clarity, we will also distinguish ISFVs which are very divergent from the other members of the genus *Flavivirus* by referring to them as ISFV^SL^. Finally, we will distinguish recently discovered viruses such as LAMV, which fall phylogenetically within the MBFV group but appear to infect only insects, as ISF-like.

The genus *Flavivirus* also includes 2 highly divergent genetic lineages not associated with any recognized group in the ninth report of the International Committee on Taxonomy of Viruses (ICTV) [7], represented by Tamana bat virus [TABV] [11] and Ngoye virus [14]. Recently, another highly divergent lineage, Mogiana tick virus [MGTV], was isolated in Brazil [24].

Whilst early interpretations of the evolutionary and taxonomic relationships within the genus *Flavivirus* have proved informative, they were restricted to some extent by the lack of complete genomic sequence data, and robust analytical methods. As a result, it has not yet proven possible to resolve the issue that phylogenies based on complete genomes and, separately, the NS3 gene show different branching characteristics from those based on the NS5 gene, despite a lack of evidence for recombination within the respective data sets [10,11,25]. In this manuscript we report the genomic sequences of fourteen flaviviruses, for which previously only limited data were available. Based on these more comprehensive datasets, we attempt to resolve hitherto unanswered questions relating to the flaviviruses, and explore frame shift characteristics. Additionally, we identify viruses that appear to have been introduced from the Old World into the New World, estimate the likely times prior to the present that these introductions occurred, and discuss factors that probably contributed to the global dispersal of these viruses.

## MATERIALS AND METHODS

### Viruses

MBFVs included in the study were all subcultured at least once in C6/36 cells. Batu cave virus [BCV], Jutiapa virus [JUTV], Phnom Penh bat virus [PPBV] and Sokuluk virus [SOKV] were amplified in the mammalian cell-line BHK21 and Sitiawan virus [STWV] was amplified in Vero cell cultures. STWV virus was kindly provided by Dr. Yuji Kono as inactivated nucleic acid in RNA-Now lysis buffer.

### Nucleic acid preparation

Viral RNA was either extracted using the BioRobot EZ1 (Viral RNA Mini kit: Qiagen) or RNA-Now (Biogentex) using the manufacturer’s recommendations. Reverse transcription was carried out using Taqman Reverse transcription reagents (Applied Biosystems) under standard conditions with random hexamers as primers.

### Polymerase chain reaction (PCR) in the conserved region of the genome

PCR was targeted at the E, NS3 and NS5 gene-conserved regions using consensus degenerate primers [8,26–28]. Sequences for the NS3 gene region were obtained using NS3-FS (5’-GGIGTIYTICAYACIATGTGGCAYGTIAC-3’)/NS3-FR (5’-TKICKICCIAYICKICCICKICKYTGIGCNGY-3’) primers in first round PCR, followed by nested PCR using X1 (5’-YIRTIGGIYTITAYGGIWWYGG-3’)/X2 (5’-RTTIGCICCCATYTCISHDATRTCIGT-3’) primers, with standard conditions and a hybridisation temperature of 45°C.

### Sequencing strategy

Specific primers were designed from the 3 conserved region sequences defined above and long-range PCR was conducted to complete the sequencing of gap between the E to NS3 genes, and NS3 to NS5 genes, using the cMaster RTplusPCR system (Eppendorf) [3]. A long PCR product sequencing protocol (LoPPs) was employed to sequence amplicons [29,30].

### PCR amplification of the 5’ and 3’ end of coding sequences

The 5’-terminal region of the genome sequence was obtained using an E-gene specific reverse primer and a forward degenerate primer in the 5’UTR designed using an alignment of available 5’ UTRs of *Culex*-spp. associated flavivirus sequences (5’-CULEX-S1: 5’-AGwiGTTCryCTGyGTGArCT-3’; position 1–21 of the Japanese encephalitis virus genome). Semi-nested PCR with a second inner virus-specific primer was also used when necessary. The 3'-terminal region of the genome was obtained using a similar strategy with virus-specific forward primers in the NS5 region and a reverse degenerate primer in the 3’UTR (3’UTR-MOS: 5’-GGTCTCCWMTAACCTCTAG-3’).

Sequencing was conducted with the primers used for amplification, or with M13 primers after cloning in a pCR2 cloning vector (Invitrogen).

Complete polyprotein open-reading frame (ORF) sequences, excluding the partial untranslated regions (UTR) regions obtained by this protocol were used for further analyses.

### Next Generation Sequencing (NGS)

Resequencing of seven of the eleven new complete polyprotein ORF sequences (AROAV, CPCV, ITV, KOUV, NTAV, TMUV and YAOV) was performed using the Ion PGM Sequencer (Life Technologies SAS, Saint Aubin, France) [31] and a random reverse transcription-amplification protocol. Reads, of minimum length 30 nucleotides, were trimmed using CLC Genomic Workbench 6.5 (QIAGEN Company), with a minimum of 99% quality per base and mapped to reference sequences previously obtained by the Sanger method. Parameters were set such that each accepted read had to map to the reference sequence for at least 50% of its length, with a minimum of 80% identity to the reference.

BCV, JUTV and PPBV sequences were obtained using the same NGS method and *de novo* assembly.

### Sequence analysis

**Fig 1:**
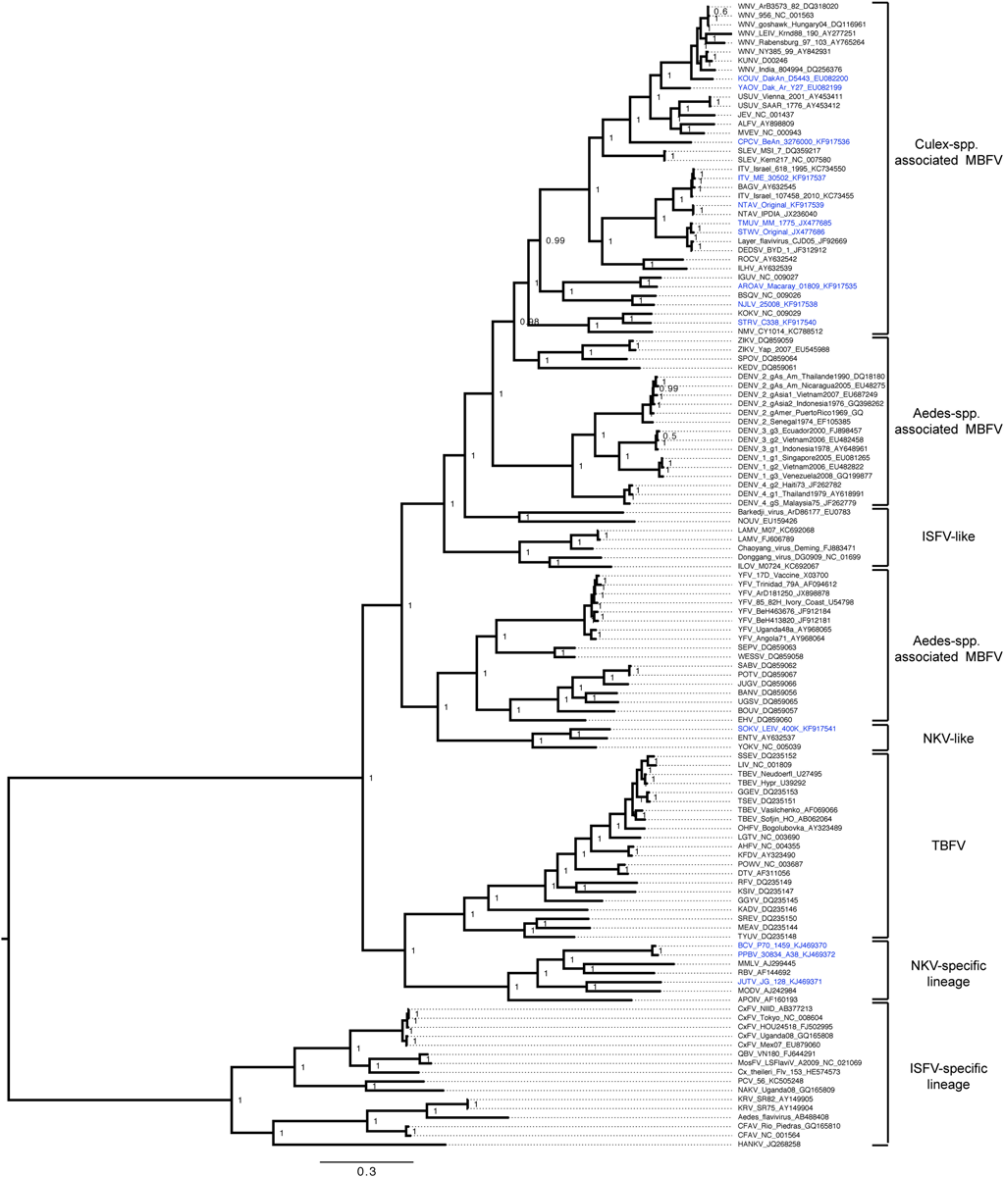
Bayesian phylogeny of the ORF ‘global genus’ amino acid dataset. Only posterior probabilities of 0.9 are included. The tree is midpoint rooted. Bar, 0.3 substitutions per site.

Sequences were refined using Sequencher 4.8 (Gene Codes, Ann Arbor, MI) and combined with other flavivirus sequences available in the Genbank database, to obtain a dataset including a representative of at least one sequence for each species available as full polyprotein ORF for the genus *Flavivirus*. Genbank accession numbers of sequences used for the analysis are noted after each virus abbreviation on Fig. 1 (tree). Complete polyprotein ORF amino acid alignments were generated using both Clustal W2 [32,33] and MUSCLE [34] available at the EMBL server (http://www.ebi.ac.uk/Tools/) and refined manually, for comparison. Nucleotide alignments were then deduced using amino acid (AA) alignments as a template using the TranAlign software available via the EMBOSS server (http://emboss.bioinformatics.nl/cgi-bin/emboss/tranalign). The effect of removing regions of ambiguous alignment via the GBlocks algorithm [35] using less stringent parameters was also investigated.

Amino acid phylogenetic trees were reconstructed using Markov chain Monte Carlo (MCMC) analysis implemented in MrBayes v3.1.2 [36]. The analysis was performed using the WAG substitution model with gamma distributed rate variation among sites and using default priors. Five independent Markov chains were run for 10 million generations, with the first 10% of samples discarded as burn-in. Stationarity was confirmed based on effective sample sizes >400 using Tracer v1.4.1 [37]. A maximum clade credibility tree was summarised using TreeAnnotator which annotates all nodes with posterior probability support values. A range of nucleotide analyses was also conducted (both including and excluding third codon positions).

Secondary RNA structures were investigated using the RNAfold webserver (http://rna.tbi.univie.ac.at/cgi-bin/RNAfold.cgi) and pknotsRG [38]. Synonymous site conservation was analysed as described previously [39].

### Bayesian inference of a time-measured evolutionary history

A second data set of polyprotein ORF flaviviruses was compiled by excluding the highly divergent sequences of all ISFVs and including additional sequences of YFV that were isolated from either the New World or the Old World. An amino acid alignment was produced using Clustal W2 and regions of ambiguous alignment were removed using the GBlocks algorithm with standard parameters.

A time-measured evolutionary history was inferred using MCMC analysis implemented in BEAST [40]. Based on model testing using Prottest, we used the LG substitution model [41] with gamma distributed rate variation among sites in conjunction with a relaxed uncorrelated lognormal molecular clock model [42] and a Bayesian skygrid tree-prior [43].

Time-calibration of the evolutionary history was based on the recognised slave-trade introduction of YFV to Brazil [12,44,45]. Specifically, based on estimates from www.slavevoyages.org, we constrained the common ancestor of the American YFVs to have existed before 1860, and the divergence from the West-African YFVs to have occurred after 1561. The divergence of the South American YFV from the African YFV strains represents the upper boundary for the introduction. Therefore, this node is assumed to be younger than 449 years. The common ancestor of all South American YFV strains represents the lower boundary for the slave trade introduction, and this node is therefore assumed to be older than 150 years. Given the long evolutionary time-scale and the fact that we performed our analysis at the amino acid level to allow the estimation of relatively deeper divergence times, instead of resorting to potentially saturated nucleotides, and to avoid a potential disconnect between short-term and long-term evolutionary rates [46], we did not consider sampling time differences when estimating the timed history.

**Fig 2:**
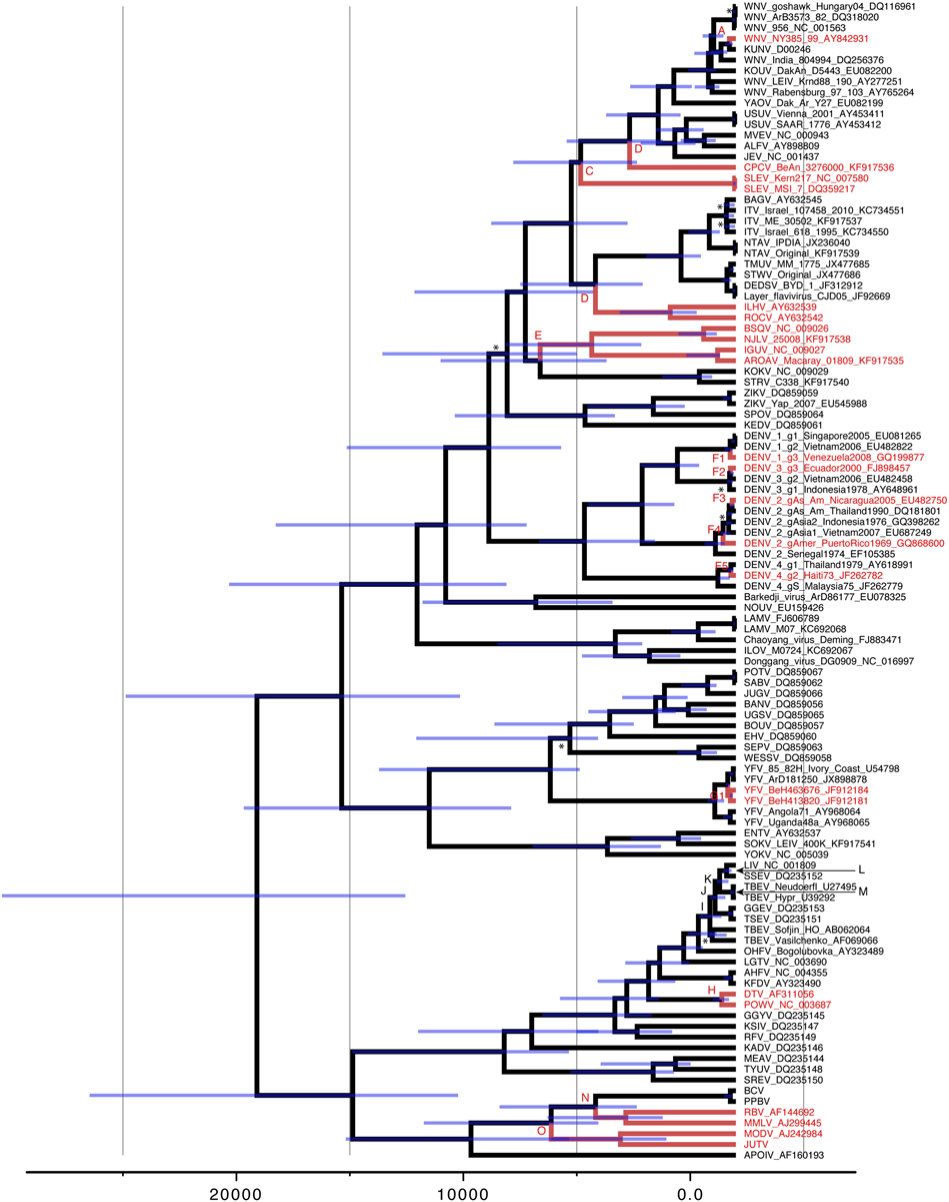
Maximum clade credibility tree summarized from the Bayesian molecular clock analysis. All nodes attained maximal probability support except for those indicated by a * (posterior probability < 0.95). The node age uncertainty is indicated using 95% highest posterior density (HPD) interval bars in blue. Old World and New World ancestral states are indicated by black and red branches/nodes respectively. Nodes of interest are listed A to N as referred in the text.

We also incorporated a two-state discrete diffusion model for the Old World and New World locations in our analyses and jointly estimated the ancestral geographical states with the evolutionary history [47]. The BEAST analysis was run for 20 million generations and diagnosized using Tracer. Trees were summarized using TreeAnnotator and visualized using FigTree (Fig. 2).

### Biogeographical data

Available ecological data, geographical dispersal patterns, host association and pathogenicity for all mosquito-borne flaviviruses were retrieved from the CRORA viral database (Centre collaborateur OMS de Reference et de Recherche sur les Arbovirus, Institut Pasteur de Dakar, Africa http://www.pasteur.fr/recherche/banques/CRORA/) and the International Catalogue of Arboviruses [48]. Three viruses tentatively assigned to the genus *Flavivirus*, TABV [11], Ngoye virus [14] and Mogiana tick [24] virus were not included in analyses because they are genetically too divergent to be incorporated without ambiguities using currently-available methods [10].

## RESULTS

Using our sequencing strategy, we obtained complete coding sequence for 10 *Culex*-spp. associated flaviviruses (Aroa virus [AROAV], Naranjal virus [NJLV], Cacipacore virus [CPCV], Koutango virus [KOUV], Yaounde virus [YAOV], Stratford virus [STRV], Israel turkey meningoencephalomyelitis virus [ITV], Ntaya virus [NTAV], Tembusu virus [TMUV] and STWV. Additionally, the remaining non-vectored viruses (NKV-like) in the MBFV group, viz., SOKV and 3 NKV^SL^ flaviviruses BCV, JUTV and PPBV were sequenced. With the addition of these new sequences, and by taking into account other recently-published data [49–52], we have produced the most comprehensive review and phylogeny of flavivirus full polyprotein ORF sequences and phylogeographic information to date.

### Phylogenetic analysis

Fig. 1 presents a phylogenetic tree based on all the sequence data yielding the highest posterior probabilities out of all analyses performed. The tree is based on amino acid sequences aligned using ClustalW2. There are high levels of support at all nodes (with a posterior probability ≥ to 0.98 for all nodes, with the exception of one node intra WNV lineage 2 at 0.6 and one node intra DENV-3 strains at 0,5). Clades suggested by this analysis correspond to the recognised virus-vector-host groups defined above and previously [1].

### TBFVs

The phylogenetic relationships of the TBFVs agree with previous studies [3] and are not elaborated upon further in this manuscript.

### NKVs

The inclusion of the complete polyprotein ORF sequence for SOKV corroborates and extends previous suggested groupings for the three NKV-like bat-associated viruses, namely Entebbe bat virus [ENTV], Yokose virus [YOKV] and SOKV which cluster within the *Aedes*-associated mosquito-borne virus clade. These three NKV-like viruses represent the ICTV “Entebbe bat virus group” [7] and form a basal lineage for the YFV group and the Edge Hill virus [EHV] group. In addition, our analysis confirms that this NKV-like group of viruses currently sits in the clade that contains *Aedes* spp. MBFVs with a robust branching pattern supported by high posterior probabilities.

Sokoluk virus was isolated in 1970 from *Vespertilio pipistrellus* bats in Kyrgyzstan [53]. According to the 9^th^ report of the ICTV [7] SOKV is a member of the Entebbe bat virus group, and appears to be a strain of the species ENTV. *Entebbe bat virus* is an African virus isolated from the insectivorous migratory bat species *Tadarida* (Chaerephon) *limbata*. It therefore seems reasonable to propose that SOKV probably emerged in Africa and dispersed to Asia via transmission across areas of overlapping bat habitats. Importantly, ENTV and SOKV have been shown to replicate in C6/36 mosquito cells *in vitro* [54] whereas the recognised NKV^SL^ that form a genetically distant clade (Rio bravo [RBV], Montana myotis leukoencephalitis virus [MMLV], etc.) do not replicate in mosquito cells [55]. This observation together with the phylogenetic position of these viruses in the MBFV clade is consistent with the idea that the transmission or maintenance cycles of YOKV, ENTV and SOKV (or ancestral representatives of these viruses), may have involved mosquitoes in the past.

The addition of the complete ORF sequences of BCV, JUTV and PPBV corroborates and extends our knowledge regarding the poorly-described NKV^SL^ group. Apoi virus (a Japanese virus isolated from *Apodemus* spp. and *Clethrionomys* spp.) constitutes a separate group which appears to have diverged earlier from the other viruses, which are distributed into two groups that include viruses isolated from rodents and bats respectively [25]. Bat-associated viruses constitute 2 sub-groups including MMLV and RBV, PPBV and BCV, respectively. Rodent-associated viruses are represented by Modoc virus and JUTV in our analysis. The other representatives of rodent and bat NKVs were not included in this analysis due to the absence of complete ORF sequences.

### MBFVs

Using partial genome sequence-based phylogenies, the MBFVs were previously divided into 2 major groups reflecting their vector associations (*i*.*e*., *Aedes-* and *Culex*-associated groups), the principal vertebrate host and associated diseases [1]. Our analysis, based on complete ORF sequence data, confirmed this division of MBFVs into 2 major epidemiologically distinct vector groups, *i*.*e*., those primarily associated either with *Culex* spp. or *Aedes* spp mosquitoes. However, additional ISFV-“like” MBFVs became available for phylogenetic analysis. Lammi virus [LAMV] was isolated from *A*. *cinereus* mosquitoes in Finland [13], Ilomantsie virus [ILOV] was isolated from *Ochlerotatus* mosquitoes in Finland (Huhtamo et *al*., submitted paper), Marisma mosquito virus [MMV] from *O*. *caspius* in Spain [18], Donggang virus [DGV] from *Aedes* mosquitoes in China (Unpublished data, GenBank acc. Number: NC_016997), Chaoyang virus [CHAOV] from *A*. *vexans nipponii* in Korea [56] and in China (Article published in Chinese, GenBank acc. Number: FJ883471), Nounané virus [NOUV] from *Uranotaenia* spp. mosquitoes in Ivory Coast [57] and Barkedji virus [BJV] from *Culex perexiguus* in Israel [58] and Senegal (Unpublished data, GenBank acc. Number: EU078325). The mosquito vector was originally unknown for CHAOV (China) and BJV (Senegal) but these viruses have also been recently described in other locations [58]. Thus, based on this more recent evidence the probable vectors for CHAOV and BJV are *Aedes* and *Culex* species respectively.

These viruses form two distinct groups (Fig. 1) that diverge from the two previously recognised groups *Aedes* spp.-associated and *Culex* spp.-associated MBFV. Moreover, the branching pattern of the tree implies that they emerged after the *Aedes* spp.-associated virus lineage but before the *Culex* spp.-associated virus lineage. Currently, they sit in the major clade of *Aedes*-associated viruses and represent an integral part of the evolutionary continuum amongst the MBFVs. These two groups of Eurasian viruses, containing LAMV, ILOV, DGV and CHAOV and the African virus group containing BJV and NOUV, appear to replicate only in mosquito cells, possibly with transitory replication in vertebrate cell lines [13,57]. This is compatible with 2 independent events during which these MBFVs lost their ability to infect vertebrate cells. Recently, Nanay virus was isolated from *Culex ocossa* in Peru [59]. This virus was only partially sequenced in the E and NS5 gene but it seems to be closely related to NOUV, leaving the question of a potentially more widespread dispersal of this new ISFV-like virus in the NW. This is not surprising, in view of other papers which reported the widespread distribution of ISFV and the huge extent of undersampling.

*Aedes*-associated flaviviruses (Fig. 1) include the dengue virus [DENV] group, the yellow fever virus [YFV] group, the EHV group, and the Kedougou virus [KEDV] group. Results from our analyses are in accordance with the most recently published studies [8].

The complete ORF sequences of 10 *Culex spp*.-associated flaviviruses were determined and included in the phylogenetic tree (Fig. 1). Thus, all viruses currently known to fall within this group have now been characterized.

#### CPCV

CPCV is a bird-associated virus, isolated in Brazil from the blood of the black-faced antbird. This virus has never been found in mosquitoes. However, this could reflect insufficient sampling of field materials. In the most recent phylogenies (Fig. 1), CPCV always roots the JEV group with a posterior probability of 1. Recently, it was isolated in Brazil from a human presenting clinically with leptospirosis and/or yellow fever-like illness [60].

#### YAOV, KOUV and WNV

YAOV and KOUV represent ancestral lineages of WNV in the phylogenetic tree (Fig. 1). YAOV has been isolated in Africa in the Cameroon, Central African Republic, Congo, Senegal and Ivory Coast from birds, mammals and both *Culex* and *Aedes* mosquito species and has never been identified as a human pathogen [61]. In contrast, KOUV was isolated in Senegal from rodents (*Tatera kempi* and *Mastomys sp*.) and also from a human following a laboratory infection [48].

The sequences of two viruses isolated in Eastern Europe and Russia, namely Rabensburg virus (RABV) and Krasnodar virus (KRDV) respectively, from a pool of *Culex pipiens* mosquitoes and *Dermacentor marginatus* ticks, were also included in the phylogenetic analysis because they are related to, but show significant divergence, from WNV [62,63]. In addition, the Indian lineage WNV strain India 804994 isolated from a human, was included in the analysis [64].

The phylogeny (Fig. 1) supports previous suggestions [65] that WNV has an African origin, as the African YAOV roots the WNV group.

#### MVEV and ALFV

Other members within the JEV serocomplex including JEV, Usutu virus [USUV], Murray Valley encephalitis virus [MVEV] and Alfuy virus [ALFV], form a strongly supported sister group to these viruses, all sharing a common ancestor with CPCV. Notably, the 2 Australian viruses MVEV and ALFV share the same ecological niche and are considered to be a single species [7], with ALFV being a strain of MVEV. In common with other JEV serocomplex viruses, MVEV and ALFV have been isolated birds and mosquitoes. MVEV causes hundreds of human cases of encephalitis annually in Australia. In contrast, there is only one unconfirmed case of mild polyarticular disease (in 1987) doe to ALFV [48]. Previous studies in laboratory animals showed that ALFV is less neuroinvasive than MVEV following peripheral challenge [66].

#### ITV and BAGV

Within the recognized Ntaya virus group [7], we have determined the polyprotein ORF sequences of NTAV, ITV, TMUV and STWV. ITV and Bagaza virus [BAGV] are bird-associated viruses that cause encephalitis in poultry and wild birds. Both of these viruses appear to have their evolutionary origins in Africa, although ITV is a frequent cause of avian disease in Israel and BAGV was recently identified as the aetiological agent of bird fatalities in birds in southern Spain [67,68]. BAGV was also isolated in India and human exposure was implied by detection of BAGV neutralizing antibodies in 15% of the human population [69]. No human exposures have been reported for ITV.

During the preparation of this manuscript, the sequences of 5 strains of ITV were reported [70]. The authors suggested that ITV and BAGV should be considered a single species with 2 different clades representing “old isolates” and “recent isolates”. The sequence presented in our analysis is an isolate from 1959, included in the clade of “old isolates” (Fig. 1) and is 6% divergent at the nucleotide level from all other ITV and BAGV sequences.

#### TMUV, STWV and DEDSV

TMUV, STWV and the recently described duck egg drop syndrome virus [DEDSV] [51] and layer flavivirus (unpublished data, Genbank acc nb.: JF926699) are also closely related southern Asian strains, mainly isolated in Thailand, China and Korea, but they are phylogenetically distinct from ITV and BAGV [71–73]. They also cause severe disease pathologies in domestic birds (chicken, duck, etc.) but have not been associated with human disease.

#### NTAV

NTAV was originally isolated in Uganda. During the preparation of this manuscript, the sequence of another strain of Ntaya virus was reported [74]. The strain provisionally designated IPD/A was collected in the Cameroon region of Africa in 1966. This virus differs by only 0.08% in nucleotide sequence when compared with the original strain that was sequenced in our study (Fig. 1). Positive human serological evidence in many regions of Africa confirms that NTAV does infect humans [74].

#### AROAV group

With determination of the ORF sequences of AROAV and NJLV, the sequences of all viruses recognised to date within the AROAV group are now known. Both AROAV and NJLV were isolated from sentinel hamsters in South America (Venezuela and Ecuador respectively). NJLV has also been isolated from *Culex* spp., mosquitoes.

Bussuquara virus [BSQV] and Iguape virus [IGUV] are 2 other representatives of this group and have been respectively isolated in sentinel monkeys and sentinel mice, in Brazil. As shown in Fig. 1, NJLV and BSQV form a sister group, as do AROAV and IGUV. These 4 viruses have all been isolated from mammals and each has its own ecological and biological niche. With the exception of BSQV which causes symptoms in humans that include fever, headache and arthralgia, other members are not known to be human pathogens.

#### STRV and KOKV

With the complete ORF determination of STRV in this study, all of the recognised viruses known to date in the KOKV group have now been sequenced. Interestingly, STRV has only been isolated from *Aedes vexans* mosquitoes whereas KOKV was also isolated from *Culex annulirostris* [48], both in Australia and Papua New Guinea. STRV is not known to cause human pathology, whereas serological evidence in humans has been reported for KOKV and occasionally it is responsible for acute polyarthritic disease (3 cases) with fever, headache and lethargy [75]. Additionally, the KOKV group also appears to include 2 new members designated TS5273 and New Mapoon virus [NMV] (CY1014). New Mapoon virus is included with complete polyprotein ORF sequence in Fig. 1 [17,76].

### ISFV^SL^

During the past decade, many new ISFV^SL^ have been isolated and their sequence data are consistent with the concept that they should be classified as a fourth major group of flaviviruses. Moreover it was recently observed that the ISFV^SL^ could be sub-divided into 2 sub-groups: *Stegomyia* (*Aedes*) associated viruses and *Culex* associated viruses based on all inferred phylogenies. With the addition of recent discoveries such as Nakiwogo virus (NAKV) isolated from *Mansonia* [20] and Palm Creek virus (PCV) isolated from *Coquillettidia* [77] the ISFV^SL^ group is becoming increasingly complex. Indeed the phylogeny now shows a potential third and/or fourth sub-group that includes NAKV and PCV. This is not surprising considering that the likely mosquito vectors of the two viruses, *Coquillettidia* and *Mansonia* spp., are considered by morphological data to be sister groups (Harbach & Kitching, 1998). Also, these viruses are significantly undersampled and more vector distribution studies and virus discovery would help to clarify their phylogenetic status.

### A timed evolutionary perspective for Old to New World introductions

From a phylogeographic point of view, the evolutionary origins and dispersal patterns of many of the flaviviruses can be tentatively deduced by considering their association between phylogenetic clustering and geographic location, knowledge of historical anthropological patterns, host/vector associations and estimated times from the present, of divergence from a common ancestor. For example, all the *Culex*-spp. associated virus clades are rooted by Old World *Aedes*-spp. associated viruses.

To provide formal support for these observations, we performed a separate Bayesian phylogenetic analysis under a relaxed molecular clock model. A summary of this analysis is represented by the maximum clade credibility tree, which has strong branching support for most of the clades; all nodes had high posterior probabilities (> 0.95) except those labelled with a star (*). Time-calibration for the tree, was based on the principle that YFV was introduced from the Old World into the New World during the slave-trade [12,44,45]. Although this provides only a single calibration point in the evolutionary history, it allows us to position other relative divergence times (Fig. 2). In this tree, viruses included in the highly divergent ISFV groups were removed to avoid the extrapolation of divergence times too far into the past, based on a relatively recent calibration.

In Fig. 2, the black nodes and branches indicate that the ancestral viral lineage was inferred to have existed in the Old World (OW) as opposed to the red nodes and branches for the New World (NW) virus lineages. Mean divergence time estimates and 95% credibility intervals (translucent light blue bars) are shown for each node in the tree. The phylogenetic estimate identifies, with high confidence, 11 independent nodes that represent introductions of mosquito-borne virus lineages (identified as A to E, F1–5 and G1–2) from the OW to the NW (Fig. 2). Table 1 summarizes the credible intervals for each node (or 95% HPD interval) and the median estimate.

**Table 1:** 

Virus	Interval low	Nodes of divergence with the common ancestor	Interval high	Fig. 2 nodes
WNV NY99 and KUNV	137	277	475	**A**
CPCV	3217	5239	7453	**B**
SLEV	4356	6928	9806	**C**
ILHV group	4107	6801	9496	**D**
AROAV group	5703	9351	13013	**E**
DENV-1 gen3 NW / OW	157	321	515	**F1**
DENV-3 gen3 NW / OW	89	186	311	**F2**
DENV-2 gen As-Am NW / OW	74	148	250	**F3**
DENV-2 gen Am NW / OW	245	450	664	**F4**
DENV-4 gen2 NW / OW	115	225	377	**F5**
YFV split OW / NW	281	385	450	**G1**
YFV NW	150	199	277	**G2**
DTV and POWV	308	601	1025	**H**
FE and Sib TBEV group and Europe TBEV group/LIV	649	1087	1610	**I**
GGEV/TSEV group and rest of Europe TBEV/LIV	465	783	1133	**J**
Europe TBEV and LIV	328	572	844	**K**
LIV and SSEV	195	369	569	**L**
TBEV Neud and TBEV Hypr	57	141	254	**M**
Bat's NKV split NW /OW	4370	7421	10414	**N**
Rodent's NKV split OW /NW	6065	9580	13750	**O**

Estimated times of divergence that belong to the “current time”, i.e., the past 500 hundred years were authenticated against historical facts relating to the slave trade and introduction of yellow fever virus into the Americas [78].

Similar estimates for divergence between OW ancestral DENV and NW strains (150 to 450 years ago) are also consistent with the concept of human introduction of these viruses into the NW via the Slave trade and other commercial exchanges (nodes F1 to F5—Fig. 2).

The divergence between Far eastern/Siberian TBEV and European TBEV is estimated to have occurred 1087 [1610–649] years ago (node I—Fig. 2)[79] and, the divergence between European TBEV and LIV/SSEV occurred about 572 [844–328] years ago (node K—Fig. 2). The estimates for LIV are consistent with historical records of the dispersal patterns of this virus in the British Isles [80].

The first outbreak of WNV in North America occurred in August 1999 in New York (http://www.cdc.gov/mmwr/preview/mmwrhtml/mm4838a1.htm). The virus was most likely introduced from North Africa [81]. In the OW, the eastward dispersal of WNV, probably via migratory birds and/or shipping, seems to have occurred many years earlier leading to the appearance of Kunjin virus (KUNV) in Australia. Fig. 2 (node A) shows that KUNV diverged from WNV approximately 277 [475–137] years ago. This corresponds quite closely with the dates of the early emigrants from the British Isles to Australia. The shipping routes included stopovers in Africa, during which KUNV, or an ancestral lineage, could have gained access to the ships via infected mosquitoes. The virus could then have been carried from Africa to Australia in these mosquitoes on the ships that transported the British emigrants.

In the Old World, Powassan virus (POWV) is found in Russia [82] and since the ancestral lineages of this virus are also OW viruses, it is reasonable to assume that POWV originated in the OW [12,83]. However, POWV is also found in the NW in Canada and the United States of America, although no other related tick-borne encephalitic viruses have been found in the New World. The ancestral lineage of POWV in the NW has diverged to produce 2 lineages [79,84] namely the current POWV lineage and a variant that adapted to deer ticks, currently referred to as deer tick virus (DTV). This is depicted in Fig. 2 as node H, where the ancestral lineage is displayed in black on the assumption of an OW origin.

There has been considerable discussion concerning the significance of these observations [79] and the hypothesis that POW was introduced into North America using the Beringian land bridge that connected Asia and North America between 15,000–11,000 years ago has recently been used to estimate modelling the temporal origin and evolution of flaviviruses (see the discussion section)[85].

Based on the phylogenetic and phylogeographic evidence, we deduce that mosquito-borne flaviviruses, corresponding to those identified at nodes A to E, F1–5 and G1–2 (Fig. 2), originated in the OW and were introduced into the New World 11 times. This does not preclude the possibility that in some cases ancestral African lineages may have dispersed to the NW before emerging as the currently recognised viruses. According to the TMCRA, these 11 events can be divided into two categories, those that have an estimated TMRCA that overlaps with the period of commercial trading by ships crossing the Atlantic Ocean, approximately during the past 500 years (YFV, node G1–2; DENV 1–4, node F1–5; WNV, node A—Fig. 2) and those for which the estimated TMRCA pre-dates this period of trading (AROAV group, node E; ILHV group, node D; SLEV, node C; CPCV, node B—Fig. 2).

On the other hand, the TMRCA estimates for the other 6 NW lineages suggest that these viruses potentially could have been introduced to the NW more than one thousand years before the initiation of historic commercial and slave trading across the Atlantic Ocean (AROAV group (5700–13000; Fig. 2 node E); ILHV group (4100–9500; Fig. 2 node D); SLEV (4400–9800; Fig. 2 node C); CPCV (3200–7500; Fig. 2 node B); NW bat NKV^SL^ (4400–10400; node N) and NW rodent NKV^SL^ (6100–13800; node O). These estimate are less accurate, due to the relatively short term calibration date we have used for YFV as reference, and could be thousands or even more years in the past. All apparent virus migrations during this period are referred to as having occurred “before the slave trade”.

Node E of Fig. 2 displays a TMRCA of 9400 years, which represents the common ancestor of the NW Aroa virus (AROAV) and Kokobera virus (KOKV) groups, i.e. groups of viruses that dispersed respectively westward to the NW and eastward to Australia, in the OW.

A similar pattern was observed at node D (TMRCA 6800 years before the present). The ILHV group emerged presumably in Africa and dispersed westward to the NW. On the other hand, the BAGV group remained in Africa, eventually emerging in Europe and the Middle East, whereas the TMUV related ancestral lineages emerged and dispersed eastward into Asia (node D—Fig. 2).

On the other hand, the JEV/USUV/ALFV/MVEV group shares a common ancestor with a TMRCA of about 2000–3000 years before the present, implying that an ancestral lineage dispersed eastwards out of Africa and appears to have dispersed to south East Asia before emerging as JEV and then dispersing widely, throughout Asia [86].

Nodes N and O in Fig. 2 identify the no known vector NKV^SL^ viruses. In common with the arboviruses, there are distinct lineages in the OW and NW (coloured black and red respectively). The TMRCA of the OW NKV, Apoi virus (APOIV), which was isolated from rodents in Japan, pre-dates all the other recognised NKV^SL^, and the MRCA of this lineage diverged to produce descendant NKV and arthropod-borne virus lineages. This leaves open the possibility that the association of flaviviruses with tick and mosquito vectors may have been an acquired trait from a non-vectored ancestral virus [25]. Nodes N and O indicate that the NKVs were introduced to the NW on at least two independent occasions. Based on the TMRCA predictions, the NKV diverged over a period of 4000 to 14,000 years ago. It has been suggested (Varelas-Wesley & Calisher, 1982) that these NKVs could have been introduced into the Americas during the Miocene/Pliocene period, possibly by migrating rodents and/or bats. This idea is consistent with the TMRCA predictions presented in Fig. 2. Nevertheless, the alternative possibility that these NW viruses emerged in the OW and were introduced more recently via rodents into the NW, following the development of trading via ships between the OW and the NW should not be ruled out. Two African viruses Dakar Bat and Bukulasa bat virus are not included in this analysis as only partial sequences for these viruses are currently available.

### A search for sequence elements associated with ribosomal-1 frameshifting

Many viruses harbour sequences that induce a proportion of translating ribosomes to shift-1 nt and continue translating in the new reading frame to produce a 'transframe' fusion protein [87]. Where functionally utilized, this is referred to as programmed-1 ribosomal frameshifting (-1 PRF). The eukaryotic-1 frameshift site typically consists of a 'slippery' heptanucleotide sequence fitting the consensus motif X_XXY_YYZ, where XXX represents any three identical nucleotides; YYY represents AAA or UUU; Z represents A, C or U; and underscores separate zero-frame codons. In the tandem slippage model, the P-site anticodon re-pairs from XXY to XXX, whereas the A-site anticodon re-pairs from YYZ to YYY, thus allowing for perfect re-pairing except at the wobble position. Certain deviations from the canonical XXX of the slippery site are tolerated in the P-site, including UCC in some members of the JEV serogroup, GGU in cardioviruses and some luteoviruses, GUU in equine arteritis virus (family *Arteriviridae*), and GGA in many ISFVs besides insect nidoviruses of the family *Mesoniviridae* and some umbraviruses and dianthoviruses. The efficiency of frameshifting depends on the identity of the slippery site nucleotides but is typically less than 1% in the absence of additional stimulatory elements. Thus, most known instances of eukaryotic-1 frameshifting are stimulated (typically to a level between 1% and 50%) by the presence of a 3' stable RNA secondary structure, such as a pseudoknot or stem-loop, that is separated from the slippery heptanucleotide by a 'spacer' region of 5–9 nt.

**Fig 3:**
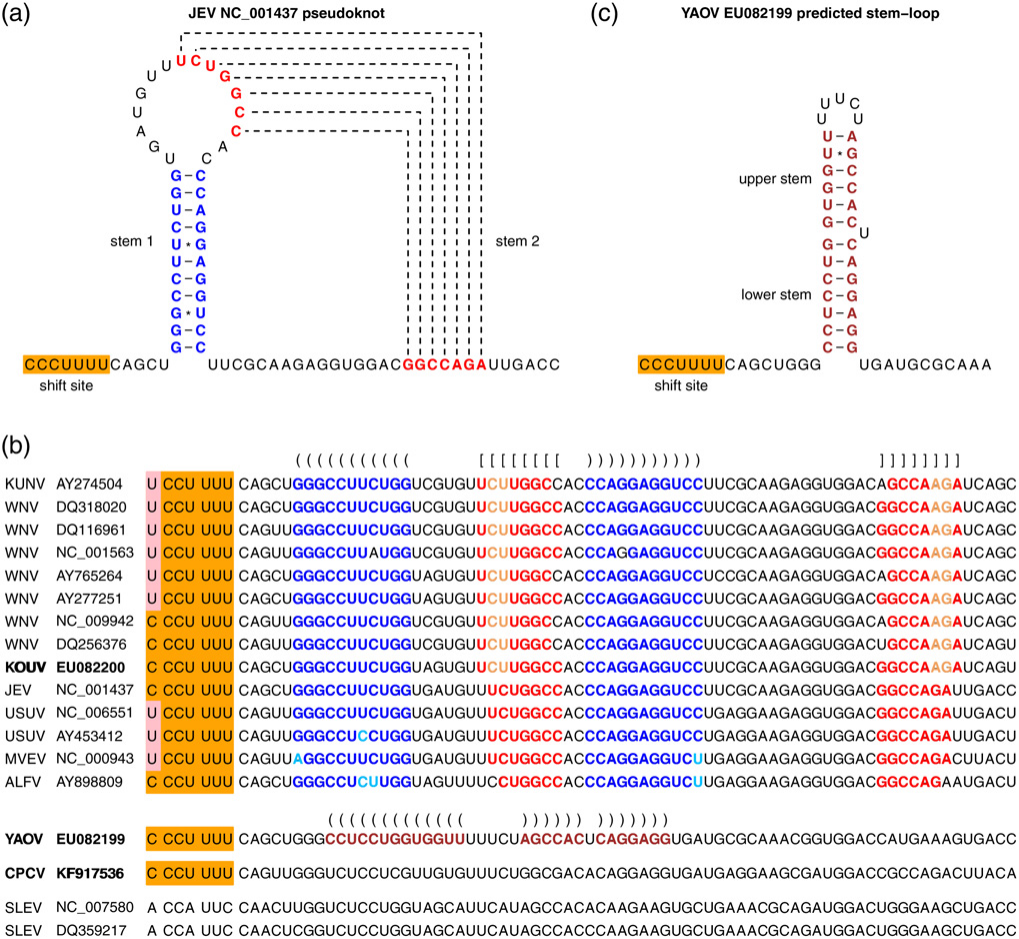
Frameshift stimulatory elements in viruses of the JEV serogroup. (a) Previously identified frameshift site (Y_CCU_UUU; Y = C or U; orange) and 3'-adjacent stable pseudoknot structure responsible for stimulating-1 PRF in the NS2A-encoding region of JEV and related flaviviruses. Stems 1 and 2 of the pseudoknot are indicated in blue and red respectively. (b) The shift site and pseudoknot are preserved in the newly sequenced KOUV but not in YAOV or CPCV. Substitutions that preserve the base-pairings in stem 1 (blue) or stem 2 (red) of the pseudoknot are indicated in pale blue and orange respectively. In YAOV, a simple stem-loop (brown) was predicted at an appropriate spacing from the shift site to act as a stimulator of-1 PRF. CPCV maintains the shift site but multiple possible 3'-proximal structures (not shown) could be predicted. SLEV sequences lack a suitable shift site at this genomic location. (c) Predicted frameshift stimulatory elements (shift site and 3'-adjacent stem-loop) in YAOV.

Sequence elements associated with-1 PRF were previously described in the JEV serogroup viruses for JEV, WNV, USUV, MVEV and ALFV [88,89](Fig. 3a). In these viruses, -1 PRF occurs (with an estimated efficiency of 20–50%) when ribosomes are positioned on the codons encoding the 8th and 9th amino acids of NS2A. When PRF occurs, ribosomes translate a 43-codon ORF in the-1 reading frame relative to the polyprotein ORF and then terminate. The resulting 52 amino acid 'transframe' polypeptide is not cleaved at the NS1|NS2A cleavage site and thus frameshifting results in the production of a C-terminally extended version of NS1, known as NS1'. Our study extends the range of viruses that possess this frameshift site to include CPCV, YAOV and KOUV. The overlapping ORF displayed a constant length (43 codons) for all viruses within the JEV serogroup, with the exception of SLEV which lacks the frameshift site.

A nucleotide alignment of the putative stimulatory elements responsible for the-1 PRF is presented in Fig. 3b. The slippery heptanucleotide Y_CCU_UUU (highlighted in orange on Fig. 3b) is absolutely conserved for CPCV, YAOV and KOUV as with other members of the JEV group except for SLEV. Predicted 3' RNA stimulatory elements are highlighted in Fig. 3b. KOUV retains the canonical stable 3' pseudoknot structure that stimulates-1 PRF in other JEV serogroup flaviviruses (Fig. 3a) [89]. In YAOV, however, a stem-loop structure was predicted instead of a pseudoknot (Fig. 3c). The CPCV sequence also lacks the potential to form the canonical JEV serogroup pseudoknot. In this case, however, several possible alternative structures could be predicted and, in the absence of experimental analysis or comparative genomic inference, it remains unclear which structure (if any) might be functionally relevant for CPCV.

**Fig 4:**
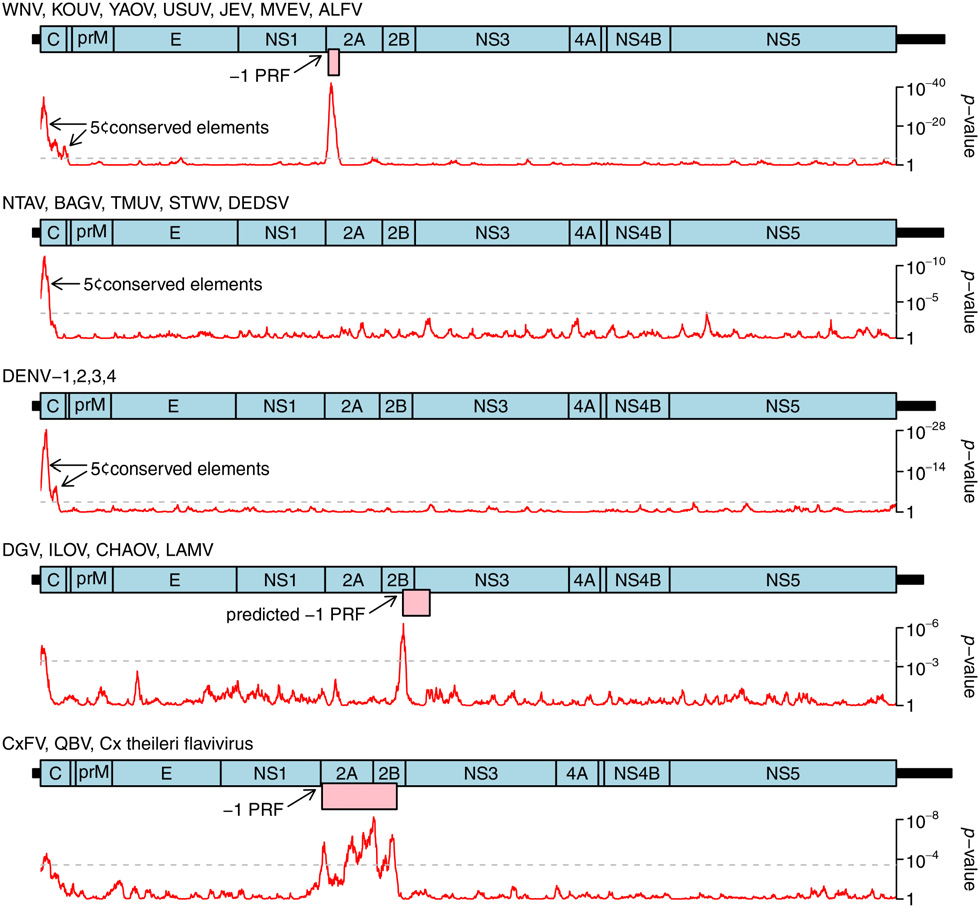
Synonymous site conservation analysis for selected flavivirus clades. Alignments of 249 JEV serogroup, 49 NTAV/TMUV clade, 89 DENV, 6 DGV/LAMV clade and 29 CxFV/QBV clade polyprotein ORF sequences were analyzed for synonymous site variability as decribed previously (Firth et al., 2011 PMID 21525127). The accession numbers of all sequences used in the analysis are available on request. Red lines indicate the probability (*p*-value) of obtaining not more than the observed number of synonymous substitutions, in a 25-codon sliding window, under a null model of neutral evolution at synonymous sites. Dashed grey lines indicate an approximate 5% false positive threshold after correcting for multiple tests (i.e. ~136 x 25-codon windows in the ~3400-codon polyprotein ORF). Statistically significant peaks in synonymous site conservation are indicative of overlapping functional elements, either coding or non-coding. Genome maps are shown for each clade. UTR lengths may be uncertain for less well-studied clades. Known and predicted overlapping ORFs accessed via-1 PRF are shown in pink. The predicted overlapping ORF in the DGV/LAMV clade is much shorter in DGV than in other members of the clade; the long form of the ORF is indicated. Note that *p*-values can not be directly compared between different clades because the statistical significance (i.e. p-value) of observed reductions in synonymous site variabilty depends on the diversity of the specific sequence alignment being analyzed.

**Fig 5:**
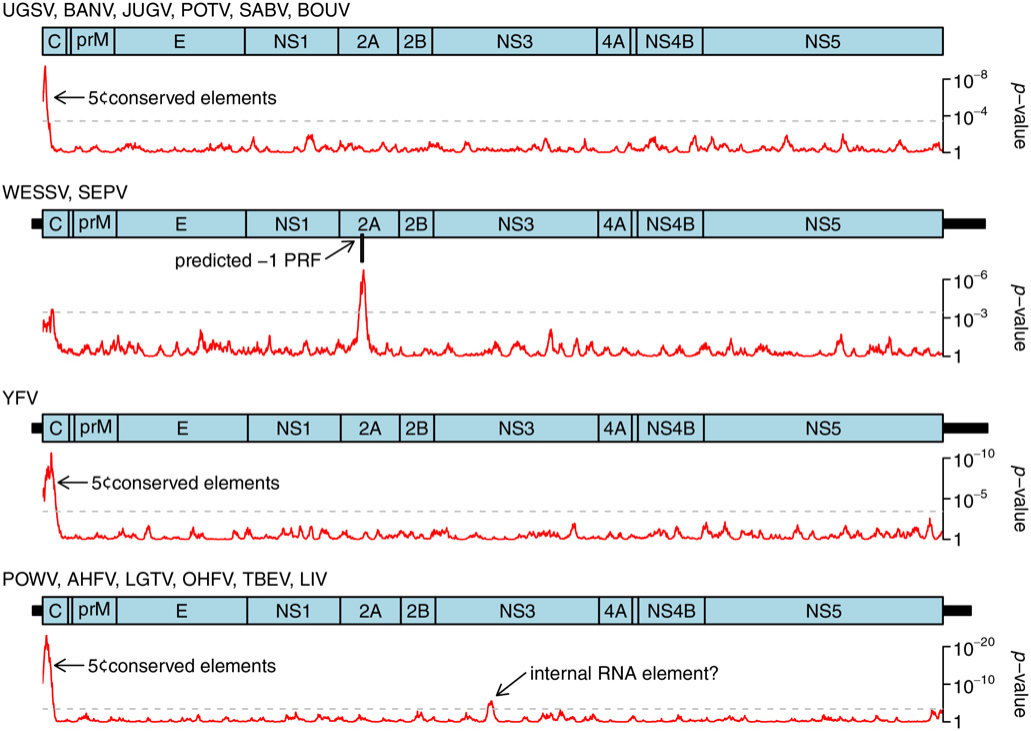
Synonymous site conservation analysis for selected flavivirus clades. Alignments of 6 UGSV/BOUV clade, 6 WESSV/SEPV clade, 56 YFV, and 144 POWV/TBEV clade polyprotein ORF sequences were analyzed for synonymous site variability as decribed previously (Firth et al., 2011 PMID 21525127). The accession numbers of all sequences used in the analysis are available on request. Red lines indicate the probability (*p*-value) of obtaining not more than the observed number of synonymous substitutions, in a 25-codon sliding window, under a null model of neutral evolution at synonymous sites. Dashed grey lines indicate an approximate 5% false positive threshold after correcting for multiple tests (i.e. ~136 x 25-codon windows in the ~3400-codon polyprotein ORF). Statistically significant peaks in synonymous site conservation are indicative of overlapping functional elements, either coding or non-coding. Genome maps are shown for each clade. UTR lengths may be uncertain for less well-studied clades and are omitted for the UGSV/BOUV clade due to lack of sequence data. Known and predicted overlapping ORFs accessed via-1 PRF are shown in pink. Note that *p*-values can not be directly compared between different clades because the statistical significance (i.e. p-value) of observed reductions in synonymous site variabilty depends on the diversity of the specific sequence alignment being analyzed.

Analysis of nucleotide conservation at synonymous sites within alignments of related flavivirus sequences has previously been successful in identifying additional coding ORFs overlapping internal regions of the polyprotein ORF and accessed via-1 PRF [88,90]. Due to the sequence constraints imposed by simultaneous coding in two overlapping reading frames, besides maintaining functional frameshift-stimulatory elements, such sites (if functionally important and phylogenetically conserved) are associated with greatly increased nucleotide conservation at synonymous sites in the polyprotein reading frame relative to the genome average. To investigate the potential presence of frameshifting in other flaviviruses, we constructed sequence alignments of selected flavivirus clades, and analyzed conservation at synonymous sites as described previously [39]. A selection of these analyses is presented in Figs. 4 and 5.

Most flaviviruses exhibit enhanced synonymous site conservation at the 5' end of the polyprotein ORF. This is presumably associated with non-coding elements such as functional RNA structures involved in replication and/or translation enhancement [91–96]. Several flavivirus clades also exhibit localized regions of statistically significantly enhanced synonymous site conservation in internal regions of the polyprotein ORF, notably the JEV serogroup, the ISFVs, the CHAOV-LAMV-DGV-ILOV clade (Fig. 4), and the WESSV-SEPV clade (Fig. 5).

In the ISFVs (CxFV-QBV clade shown in Fig. 4), enhanced synonymous site conservation is apparent in the NS2A/2B-encoding region; this corresponds to a long overlapping ORF that is accessed via-1 PRF [90]. Many more ISFV sequences are now available, and the overlapping ORF is conserved in all except for the cell culture adapted original isolate of CFAV (GenBank acc. M91671) [23]. The overlapping ORF ranges from 270 to 293 codons in CxFV, QBV, PCV, NAKV and *Cx theileri* flavivirus, and 253 to 257 codons in KRV, CFAV, HANKV, AEFV and *Ochlerotatus caspius* flavivirus. The frameshift site generally appears to be G_GAU_UUY (Y = U or C), with notable exceptions appearing to be G_UUU_UUU in NAKV and A_AAU_UUU_UUC (potential tandem shift sites) in PCV.

In the CHAOV-LAMV-DGV-ILOV clade enhanced synonymous site conservation is apparent in the region encoding NS2B. This is associated with a conserved G_GAU_UUU slippery heptanucleotide and a 3'-adjacent predicted stem-loop structure that stimulates-1 PRF in dual reporter assays and whose functionality is supported by compensatory substitutions (i.e. paired substitutions that preserve the predicted base-pairings) [52,90]. Here, the putative frameshift ORF has variable length (107 codons in LAMV and CHAOV, 71 codons in ILOV, but only 6 codons in DGV)[52], perhaps explaining why the conservation peak does not extend throughout the 107-codon ORF annotated on the genome map in Fig. 4.

**Fig 6:**
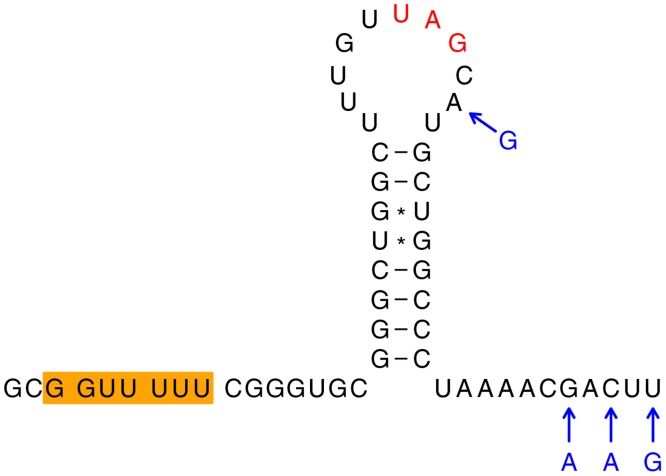
Predicted frameshift stimulatory elements in WESSV and SEPV. Frameshifting is predicted to occur on a conserved G_GUU_UUU heptanucleotide (orange) in the NS2A-encoding region, stimulated by a 3'-adjacent stem-loop structure. The-1 frame stop codon is indicated in red. The WESSV RefSeq NC_012735 is shown; nucleotide differences in the SEPV RefSeq NC_008719 are indicated in blue.

A dramatic and statistically significant peak in synonymous site conservation in an internal region was observed in one other clade of flaviviruses—the WESSV-SEPV clade (Fig. 5). This could represent an overlapping non-coding RNA element or yet another-1 PRF site. Inspection of the sequences corresponding to the conservation peak revealed a conserved slippery heptanucleotide, G_GUU_UUU [the same shift site that is utilized for-1 PRF in cardioviruses and some species of luteovirus, besides both-1 and-2 PRF in porcine reproductive and respiratory syndrome arterivirus [97–99]], and the potential for a 3'-adjacent stem-loop to form at the appropriate spacing potentially to act as a stimulator of-1 PRF (Fig. 6). In this case the overlapping ORF has just 7 codons, with the termination codon embedded within the predicted stem-loop structure. Frameshifting here would result in a greatly truncated version of the NS2A protein with a distinct 7 amino acid C-terminal end encoded by the short overlapping ORF.

A statistically significant peak in synonymous site conservation was also observed in the NS3-encoding region of TBEV and related flaviviruses (Fig. 5). However this was not thought to represent a PRF site, first, because the degree of conservation was relatively modest compared to what is typically observed at a PRF site, and, second, because we were unable to identify a canonical PRF-compatible shift site at this genomic location in these viruses. Instead, it appears to correspond to a small conserved RNA stem-loop structure.

Internal frameshift sites and/or overlapping ORFs were not predicted using these methods for other flavivirus groups such as SLEV, YFV and DENV. It should be noted, however, that the synonymous site conservation analysis would not necessarily detect cases of frameshifting where there is not an overlapping ORF subject to purifying selection and where the frameshift stimulatory elements comprise just a few codons (e.g. primary sequence rather than secondary structure stimulators) and/or are located in the nascent peptide rather than the RNA sequence. Also these analyses do not yet provide statistically useful results for all flavivirus clades (e.g. where there is insufficient sequence divergence within a clade and too much sequence divergence between phylogenetically adjacent clades to observe the effects of purifying selection at synonymous sites).

## DISCUSSION

As phylogenetic methods have developed and more complete genome (ORF) sequence data have become available, increasingly robust analyses and interpretations of the flaviviruses have become possible. For example, we now know that the African virus, Kadam virus (KADV), sits at the root of the TBEV lineages and KADV diverged from a common ancestor of the seabird tick-associated viruses (Fig. 1).

Our phylogeny strongly supports the NS3 branching pattern of divergence defined previously [10,11,25] in which the TBFVs and NKVs share a common ancestor. The analysis confirms previous suggestions that the *Culex*-associated flaviviruses evolved from ancestral *Aedes*-associated viruses [8,12]. However, the recent discovery of several new viruses included in the ISFV-like group, now reveals the possibility of two potentially new virus groups, viz., the LAMV group and the NOUV group. They are geographically very widely dispersed but the mechanisms for their dispersal are unknown. Currently exclusively OW viruses found in Africa, Europe or Asia have been characterised. However, with the addition of the newly isolated Nanay virus in Peru [59], which is closely related to NOUV, the ISFV-like group now seems to extend to the NW and this will need to be re-visited when genomic sequences of NW isolates become available.

These recently-discovered apparently non-pathogenic mosquito-associated viruses (LAMV and NOUV group) are phylogenetically closely-related to human pathogenic viruses, such as DENV, YFV, WNV, JEV. However, they do not appear to replicate in vertebrate cells [13,52,57] a property which to date has precluded the possibility of isolating such viruses from field material by intracerebral inoculation of newborn mice. Possibly, they represent independent lineages of viruses that, like the NKV, have lost a particular genetic trait not required for their long-term survival.

Within the MBFVs, three NKV-like viruses, ENTV, SOKV and YOKV, have not been associated with any known vector. Nevertheless, they are genetically closely related to the YFV and EHV groups, have only been found in the Old World, and are associated with migratory bats. YOKV and SOKV were both isolated in Asia but appear to have their ancestral roots in Africa, as do many of the related viruses in the EHV group. Thus, flaviviruses seem able to lose or acquire a variety of genetic traits as illustrated by acquisition of the requirement for a tick or mosquito vector and therefore becoming arboviruses, loss of ability to infect vertebrate cells, exemplified by LAMV and NOUV, loss of the requirement for vector transmission illustrated by the ENTV group and originally proposed by Mattingly in 1960 [100] and supported by Kuno and Chang [101], inability to infect vertebrate cells, as illustrated by the ISFVs and acquisition of a frameshift as typified by the JEV serogroup viruses (discussed in detail later).

### No known vector flaviviruses specific lineage (NKV^SL^)

In contrast with the MBFV and TBFV groups, the NKV^SL^ group remains poorly described. Based on the phylogenetic and TMRCA data, viruses within this group have been introduced to the NW on at least two separate occasions and these introductions may have occurred thousands of years ago. Estimations for the time of divergence between these OW and NW NKV^SL^ ranged between 4000 to 14000 years ago (Fig. 2 nodes N and O). Despite the recognised large errors in TMRCA estimates, they all pre-date, by thousands of years, the recognised commercial trading period across the Atlantic Ocean that commenced 400–500 years ago. Therefore, viruses could have dispersed to the NW from the OW thousands of years before transoceanic trading was taking place. The possible mechanisms of dispersal remain a mystery. It is now established that OW human populations (presumably of both Asian and European origin) became established in the Americas during the period of TMRCA estimates, and possibly even before these estimated times. The question arises, during such migrations is it likely that infected animals could have been transported (*e*.*g*., rodents). The arrival of viruses in the NW could alternatively have occurred via the gradual dispersal of OW rodents and migratory bats. On the other hand, these transoceanic dispersions might have occurred after the development of trading between the OW and the NW.

### Insect-specific flaviviruses (ISFVs)

Until about ten years ago, only one ISFV, cell fusing agent virus (CFAV), was recognised. Currently, at least 9 genetically distinguishable ISFVs have been isolated and their sequences determined. This group of mosquito- and potentially sandfly-borne viruses is an extremely genetically diverse group with a divergence of up to 61% at the amino acid level. This compares with the genetic distances between the three current genera in the family *Flaviviridae*. Moreover, they infect only invertebrate hosts and DNA forms of ISFV genomes are generated during infection of cell cultures [26]. Integrated DNA sequences have been identified in mosquito genomes and isolated DNA forms have been detected in field samples [18,26,102,103]. Currently, there is no clear indication of the biological significance of these DNA forms amongst ISFVs. We therefore propose that the distinct differences of the ISFVs justify their inclusion as a separate genus in the family *Flaviviridae*. These viruses are also interesting because similar ISFVs have been isolated from mosquito species that inhabit different ecological niches, raising the question how might this occur? Possible mechanisms of virus transfer between mosquito species include the diffusion of viruses between larvae at sites shared by different mosquito species, or biting midges could become infected when taking blood meals from infected mosquitoes [104–108] and they could then act as vectors of the virus if they subsequently feed on different mosquito species.

### A perspective on Old World to New World flavivirus introductions

It is recognised that YFV and DENV were transported frequently to the Americas from Africa on the ships that transported slaves across the Atlantic Ocean during the centuries of slave trading [44,45,78,109–111]. Based on this assumption, we estimated divergence times and ancestral OW/NW relationships to provide a deeper understanding of the evolution and dispersal patterns of flaviviruses. Since virus evolution characterised by lineage-specific and non-constant substitution rates (Pettersson 2014), it would be unwise to assert that a single calibration point can produce precise estimates for both short/recent times, and long/deep times. Not surprisingly, our estimates, based on a calibration point from the recent historical period and involving mosquito-borne viruses, differ significantly from those of Pettersson and Omar—whose calibration point was derived from a more distant historical time period and involved a tick-borne virus. An important consequence of the latter calibration is the hypothetical origin of mosquito-borne flavivirus diversification during the last glaciation period, whilst the former calibration suggests that the major part of mosquito-borne flavivirus diversification which involves human epidemiology, occurred after the end of the most recent major Ice Age [12].

Our choice was to use an ‘independently confirmed’ historical hypothesis [44] and to exclude from analysis highly divergent viruses for which genomic and biological information remains scarce (insect specific viruses, Tamana bat virus, Ngoye virus, Mogiana tick virus. . .). Therefore, based on these assumptions our estimates are more accurate for the recent historical period than for the deepest nodes of the trees, and for mosquito-borne viruses than for tick-borne or no-known vector flaviviruses.

Our results demonstrate that such movement of arboviruses from Africa to the Americas is not unique to YFV and DENV. The ancestral history presented in Fig. 2 provides clear indications that multiple introductions of viruses have occurred from the OW to the NW during relatively recent millenia. All of the *Culex*-associated viruses that circulate in Europe, Asia, Australia and/or the Americas, appear to have their evolutionary roots in Africa. Given the OW diversity of flaviviruses and the comparatively lower number of NW viruses, this analysis attempts to formalise a parsimonious argument for an ‘Out-of-Africa’ history. Unless there is a matching, but largely unsampled flavivirus diversity in the NW, this remains the most plausible interpretation of their phylogenetic distribution.

Given that we rely on a single, relatively recent calibration to estimate old divergence times, we do not consider our estimates to be a precise historical record of flavivirus evolution, but it does allow us to distinguish 2 major temporal periods during which flaviviruses may have been introduced from the OW into the NW. All historical and phylogenetic data are consistent with the concept that there was a recent period of 400 to 500 years during which African viruses such as YFV and DENV were transported on slave and commercial ships across the Atlantic Ocean to the Americas, [12,44,45,78,111,112] which is why we base our estimates on this for the divergence time estimation. This “recent evolutionary period” includes viruses that have gradually emerged during the past few decades viruses (e.g., WNV, HIV, SARS, CoV, HepC virus, USUV, MERS CoV) and are still being dispersed via human movement and commercial transportation.

Based on published estimates for the times of divergence in the tree [9,44,113,114] it also appears that these introductions to the New World may have occurred gradually over the period of time corresponding to the movement of humans from Africa to the New World during the four or five hundred years of the Slave trade. Support for the concept that DENV can be included with YFV also comes from the known presence of the American genotype dengue viruses in South America that have African ancestral lineages. These viruses were presumably also transported to the Americas over the same range of time as the introduction of yellow fever viruses [12] and even more recently as slave trading was finally abolished [115]. It has been recorded that febrile syndromes and even haemorrhagic fever, clinically compatible with dengue fever, were diagnosed clinically in southern parts of North America over a long period of time [111,116,117]. The most recent and persuasive example of an OW introduction to the NW was the spectacular appearance of West Nile virus (WNV) in New York in August 1999 [118]. Since the first cases of WNV encephalitis in birds and humans were discovered in the area of the Bronx zoo and relatively close to a major international airport, it is considered possible that WNV was inadvertently introduced into North America either via infected birds or mosquitoes transported to the New York area by air transport [12,65,119].

Fig. 2 also identifies 4 examples of other flaviviruses (CPCV—node B; SLEV—node C; ILHV group—node C; AROAV group—node E) that have been introduced from the OW to the NW. However, the TMRCA for these introduced viruses predates the period of slave trading, by thousands of years and will be referred to as “ancient period” viruses.

If we consider the possibility of a more ancient introduction of viruses, i.e. between 4000 to 14000 years ago, we have to consider the possibility that ancestral viruses emerged in the OW and were then introduced into the NW many years before the slave trade period, possibly by birds, rodents, bats, arthropods and/or humans. The tree shows that introductions to the NW were multiple and independent. The first introduction is represented in Fig. 2—node E for the AROAV group. Except for BSQV, these viruses have only been isolated from rodents. The TMRCA for this node is 5700 to 13000 years ago. An ancestral virus present in the OW could have been introduced into the NW via rodents or migratory birds and then adapted to other species such as rodents. Other predicted introductions are presented in Fig. 2 nodes-C, B and D for ILHV/ROCV, SLEV and CPCV. These viruses are bird associated and may therefore have been introduced via migratory birds or bats. However, if birds were a major cause of virus introduction into the NW, it seems surprising that viruses such as WNV have only been successfully introduced once, as suggested by the numerous phylogenetic analyses of many North America WNV isolates [81,120,121]. Additionally, Nanay virus isolated in *Culex* mosquitoes in Peru might represent another example of potential virus introduction from the OW to the NW.

Recent discoveries of viruses in the ISF group such as Culex flavivirus [20,21,122,123] show that *Culex* spp.-associated viruses contain members from both the OW and the NW. It has been proposed that in the past, several different insect-specific flaviviruses have been introduced independently into Latin America, from the OW, rather than a single virus having been introduced with subsequent divergence to generate the different viruses found in the Americas [49,124].

The NKV^SL^ Apoi virus, an OW virus isolated in Japan from *Apodemus* mice (*Muridae* family, *Murinae* sub-family) roots all other NKV^SL^. MODV and JUTV were the first recognised rodent NKV^SL^ to be introduced to the NW (Fig. 2—node O) about 9500 years ago. MODV was isolated in North America from *Peromyscus* mice (*Muridae* family, *Sigmodontinae* sub-family) whereas JUTV was isolated in South America. These viruses were possibly introduced into the NW via rodents, when the OW and NW land masses in the northern hemisphere were joined by ice.

Rio Bravo virus (RBV) and Montana myotis leukoencephalitis virus (MMLV, represent a second introduction of NKVs into the NW (Fig. 2—node N). Their ancestral lineage appears to have diverged from an ancestral lineage of the OW bat NKVs.

Studies on Arenaviruses and Hantaviruses originally led to the development of the concept that viruses have co-evolved with their rodent hosts over time-scales of millions of years during which the viruses were assumed to have been transported during the gradual introduction of rodents from the OW into the NW [125–127]. However more recent studies do not appear to support this concept since for hantaviruses, estimates of virus divergence are in the order of thousands of years, as they exhibit short-term substitution rates of 10–2 to 10–4 substitutions/site/year [128,129]. It is hence more likely that Hantaviruses adapted relatively recently to their rodent hosts.

In the case of rodent-associated flaviviruses, and particularly NKV^SL^, only limited genetic data are available and due to presumed undersampling of viruses, to date there is no clear indication of the exact period of introduction of viruses to the rodent species in the NW. However, by analogy with the hantaviruses, and taking into account the TMRCA presented in Fig. 2, it seems unlikely that flaviviruses have coevolved with their rodent hosts over millions of years.

Although the uncertainty in the TMRCA is quite significant, there are clearly two periods of emergence, *i*.*e*. an “ancient period” in the order of magnitude of thousands of years ago, and a more “recent” period in the order of decades to a few hundred years ago. We note that it is important to be aware of the fact that estimates for the “ancient period” including estimates for NK^SL^, are based on a single calibration event of an *Aedes* vectored virus (YFV) during the “recent period”. This is almost certainly not an accurate estimation of the evolutionary dynamics that occurred during the “ancient period”, which in the case of the NKV^SL^ are not vectored by arthropods.

### Frameshifting in the MBFVs and ISFVs

It was previously reported that members of the JEV serocomplex express a transframe fusion protein, NS1', via-1 PRF [89]. The frameshift-stimulatory elements—a 'slippery' heptanucleotide sequence and the potential to form a stable 3'-adjacent pseudoknot structure—are conserved in all recognised members of the JEV serocomplex with the exception of SLEV [88]. However, the sequences of CPCV, YAOV and KOUV had not been determined when this conclusion was reached. We now show that the frameshift site is also conserved in these newly sequenced viruses. Thus, the ability to produce NS1' via PRF appears to have been acquired as a genetic trait after the branching point which separates the New World virus SLEV (Fig. 2—node C) from another New World virus (CPCV) and the remaining Old World JEV serocomplex viruses (Fig. 2—node B).

The CPCV sequence also lacks the potential to form the canonical JEV-serogroup 3' RNA stable pseudoknot that is associated with highly efficient-1 PRF (20–50%; [89]); thus it is possible that frameshifting in CPCV is relatively inefficient.

The significance of the NS1' protein is not yet fully understood but it has been reported that it plays a role in viral neuroinvasiveness and reduced neurovirulence [89,130,131].

Frameshifting at a very similar genomic location occurs in the ISFVs but results in the translation of a much longer overlapping ORF [90]. Potential frameshift sites have also been bioinformatically predicted for NOUV, CHAOV, LAMV and KEDV (Firth *et al*., 2010); ILOV and DGV [52]; and SEPV and WESSV (see above). These potential frameshift sites are also located within the genomic region encoding NS2A/NS2B. Thus, the acquisition of PRF at internal regions of the polyprotein ORF appears to be a common (though not ubiquitous) theme of flavivirus evolution. Aside from the ability to produce new functional proteins, where PRF is efficient, it may also play a role in downregulating production of the 3'-encoded replicative proteins and more quickly recycling the host cell translational machinery for increased production of the 5'-encoded structural proteins.

In conclusion, with the addition of 14 new flavivirus ORF sequences, the estimation of phylogenies via Bayesian methods, plus biogeographic and bioinformatic considerations, we have identified 11 likely introductions of mosquito-borne flaviviruses from the OW to the NW over two separate temporal periods. We have also demonstrated that similar introductions have occurred eastwards from Africa to Australia, again over two distinct time periods. Clearly there have been far more introductory movements of flaviviruses from one part of the world to another than we have described here. However, more data for a larger number of samples will be required before we can draw specific and more detailed conclusions. Finally, in the context of flavivirus evolution and dispersal, we have extended our current understanding of frameshifting amongst the flaviviruses.
